# Treatment intensification with hepatic arterial infusion chemotherapy in patients with liver-only colorectal metastases still unresectable after systemic induction chemotherapy – a randomized phase II study -- SULTAN UCGI 30/PRODIGE 53 (NCT03164655)- study protocol

**DOI:** 10.1186/s12885-020-6571-7

**Published:** 2020-01-30

**Authors:** Alice Boilève, Aline Maillard, Mathilde Wagner, Clarisse Dromain, Christophe Laurent, Eric Dupont Bierre, Samuel Le Sourd, Franck Audemar, Ayhan Ulusakarya, Veronique Guerin-Meyer, Denis Smisth, Veronica Pezzella, Thierry De Baere, Diane Goere, Maximiliano Gelli, Julien Taieb, Valérie Boige

**Affiliations:** 1 Department of Medical Oncology, Gustave Roussy, 114 rue Edouard Vaillant, 94805 Villejuif Cedex, France; 2 Department of statistics and epidemiology, Villejuif, France; 3 Centre for Research in Epidemiology and Population Health (team 2), INSERM U1018, Paris-Saclay University, Villejuif, France; 4 Department of radiology, CHU Pitié Salpétrière, Paris, France; 5 Department of radiology, Centre Hospitalier et Universitaire Vaudois, Lausanne, Switzerland; 6 Department of hepatogastroenterology, Hôpital Haut Levêque, Pessac, France; 7 Department of digestive surgery, CHP Saint Grégoire, Saint-Grégoire, France; 8 Department of medical oncology, Centre Eugène-Marquis, Rennes, France; 9 Department of hepatogastroenterology, Centre hospitalier Côte Basque, Bayonne, France; 10 Department of medical oncology, Hôpital Paul Brousse, Villejuif, France; 11 Department of medical oncology, Institut de Cancerologie de l’Ouest, Angers, France; 12 R&D Unicancer, Paris, France; 13 Department of interventional radiology, Gustave Roussy, Villejuif, France; 14 Department of Surgical Oncology, Hôpital Saint Louis, Paris, France; 15 Department of Surgical Oncology, Gustave Roussy, Villejuif, France; 16 Department of digestive oncology, Hôpital Européen Georges-Pompidou, Sorbonne Paris Cite/Paris Descartes University, Paris, France

**Keywords:** Colorectal cancer, Liver metastases, Liver resection, Hepatic arterial infusion, Oxaliplatin, Randomized trial

## Abstract

**Background:**

Approximately 40% of colorectal cancer patients will develop colorectal liver metastases (CRLM). The most effective approach to increase long-term survival is CRLM complete resection. Unfortunately, only 10–15% of CRLM are initially considered resectable. The objective response rates (ORR) after current first-line systemic chemotherapy (sys-CT) regimens range from 40 to 80% and complete resection rates (CRR) range from 25 to 50% in patients with initially unresectable CRLM. When CRLM patients are not amenable to complete resection after induction of sys-CT, ORRs obtained with second-line sys-CT are much lower (between 10 and 30%) and consequently CRRs are also low (< 10%). Hepatic arterial infusion (HAI) oxaliplatin may represent a salvage therapy in patients with CRLM unresectable after one or more sys-CT regimens with ORRs and CRRs up to 60 and 30%, respectively. This study is designed to evaluate the efficacy of an intensification strategy based on HAI oxaliplatin combined with sys-CT as a salvage treatment in patients with CRLM unresectable after at least 2 months of first-line induction sys-CT.

**Objectives and endpoints of the phase II study:**

Our main objective is to investigate the efficacy, in term of CRR (R0-R1), of treatment intensification in patients with liver-only CRLM not amenable to curative-intent resection (and/or ablation) after at least 2 months of induction sys-CT. Patients will receive either HAI oxaliplatin plus systemic FOLFIRI plus targeted therapy (i.e. anti-EGFR antibody or bevacizumab) or conventional sys-CT plus targeted therapy (i.e. anti-EGFR or antiangiogenic antibody). Secondary objectives are to compare: progression-free survival, overall survival, objective response rate, depth of response, feasibility of delivering HAI oxaliplatin including HAI catheter-related complications, and toxicity (NCI-CTCAE v4.0).

**Methods:**

This study is a multicenter, randomized, comparative phase II trial (power, 80%; two-sided alpha-risk, 5%). Patients will be randomly assigned in a 1:1 ratio to receive HAI oxaliplatin combined with systemic FOLFIRI plus targeted therapy (experimental arm) or the best sys-CT plus targeted therapy on the basis of their first-line prior sys-CT history and current guidelines (control arm). One hundred forty patients are required to account for non-evaluable patients.

**Trial registration:**

ClinicalTrials.gov, (NCT03164655). Trial registration date: 11th May 2017.

## Background and rationale

### Secondary resection of colorectal liver metastases after systemic chemotherapy

Approximately 40% of colorectal cancer (CRC) patients will be diagnosticated with colorectal liver metastases (CRLM), either at primary tumor diagnosis (20%) or during disease progression (20%) [1–4]. The only chance of cure and the most effective approach to increase long-term survival is complete CRLM resection, with a 5-year overall survival (OS) rates between 30 and 40% [5–7]. Unfortunately, only 10–15% of CRLM are initially considered easily resectable. Therefore, induction systemic chemotherapy (sys-CT) to shrink the tumor is often required to convert unresectable to resectable CRLM allowing resection with curative intent and a favorable long-term prognosis, with a 33% 5-year survival rate [8].

In selected patients with liver-only metastasis, complete resection (or ablation) rate (CRR) is reported to be linearly proportional to the objective response rate (ORR) [9]. Current first-line sys-CT regimens combining fluorouracil and oxaliplatin, with or without irinotecan, associated with targeted therapies (anti-EGFR or antiangiogenic antibodies) achieve ORR ranging from 40 to 80% [7] with (R0-R1) CRR ranging from 25 to 50% in patients with initially unresectable CRLM [10–12].

When CRLM patients are not amenable to resection after induction sys-CT, ORRs obtained with second-line sys-CT are much lower, between 10 and 30% and consequently CRRs are expected to be low (< 10%) in this population of patients [9]. The CRRs following modern second-line sys-CT regimens have not been prospectively assessed. In a retrospective post-chemotherapy hepatectomy study, published by Adam et al. [10], only 7% of patients underwent curative hepatectomy after second-line treatment.

### Hepatic arterial infusion of chemotherapeutic agents

Since liver metastases mainly receive blood supply from the hepatic artery whereas normal liver tissue is primarily perfused by the portal vein, hepatic arterial infusion (HAI) was developed to increase the local concentration of cytotoxic agents to liver metastases. As a result, HAI achieved significantly higher tumor response rates with limited systemic toxicity compared to sys-CT in patients with unresectable CRLM [13–22]. A significant impact on OS has been inconstantly observed in previous randomized trials with HAI fluorodeoxyuridine (FUDR) or 5-Fluorouracyl (5-FU), mainly due to extra-hepatic disease and/or the design of these studies that allowed cross-over of HAI [23].

Several chemotherapeutic agents have been administered via HAI to treat CRLM [24]. FUDR is mainly used for HAI because of its short half-life (< 10 min) and extensive extraction/metabolism during the first liver passage (94–99%) [21, 24, 25]. However, the biliary related-toxicity and the inconvenience of a surgically implanted port requiring a 2-week continuous infusion are the main limitations of HAI FUDR. To improve the tolerance and efficacy of HAI FUDR, the addition of steroid agents in the hepatic artery (in order to reduce biliary toxicity) and addition of “modern” concomitant systemic cytotoxic drugs (such as irinotecan or oxaliplatin) has been developed [26, 27].

Alternatively, more recent HAI chemotherapeutic agents can be used, of which oxaliplatin is one of the most important. We reported that HAI oxaliplatin accumulates in liver metastases with a concentration ratio tumor/normal parenchyma of 4.3 with a significant decrease of total platinum and filtrable platinum [28]. This suggests an improved benefit of the HAI route in terms of tolerance (e.g., reduced peripheral neuropathy and hematological toxicity) and efficacy. HAI oxaliplatin also presented a liver extraction ratio of 0.47 [29] and is more convenient than FUDR (2-h HAI of oxaliplatin repeated every 2 weeks; continuous HAI of FUDR during 14 days repeated every 4 to 5 weeks). In addition, we reported that HAI oxaliplatin overcomes resistance to prior intravenous (IV) oxaliplatin in heavily pretreated patients [30].

Our previous multicentric phase II trial showed that HAI oxaliplatin and systemic 5-FU-folinic acid (LV5FU2 schedule) induced a response rate of 64% (95% confident interval: 44–81%) with a median survival of 27 months (survival rates were 82% at 1 year and 63% at 2 years) in 28 patients with unresectable CRLM in the first-line (*n* = 7) or second-line (*n* = 21) settings [30]. The toxicity was manageable including mostly grade 3 (*n* = 8) and grade 4 (*n* = 2) neutropenia, as well as severe abdominal pain during oxaliplatin administration (*n* = 6).

In a retrospective study, we showed that adding HAI oxaliplatin to systemic 5-FU in 87 patients (78% after one or more lines of sys-CT) successfully converted unresectable to resectable CRLM in 24% of patients, with a complete pathological response rate of 19% in patients who underwent surgery [31].

Lastly, the OPTILIV prospective multicenter phase II study assessed HAI triplet chemotherapy (5-FU, oxaliplatin, and irinotecan) combined with systemic cetuximab in 64 heavily pretreated patients with KRAS wild-type CRC with unresectable CRLM after at least one first-line sys-CT, of which more than 50% had received > 2 lines of chemotherapy [32]. The ORR was 40.6% and the CRR (R0-R1) was 29% [32].

To date, no randomized study has compared HAI combined with sys-CT to standard systemic regimens in patients with CRLM still unresectable after ‘modern’ induction sys-CT. Furthermore, we have shown that the development of percutaneous hepatic arterial catheter insertion under radiology guidance have greatly increased the feasibility, functionality, and safety of HAI chemotherapy [33, 34]. We have thus designed a study to evaluate the efficacy of an intensification strategy based on HAI oxaliplatin combined with sys-CT as a salvage treatment in patients with CRLM unresectable after at least 2 months of induction sys-CT.

## Methods

**Fig. 1:**
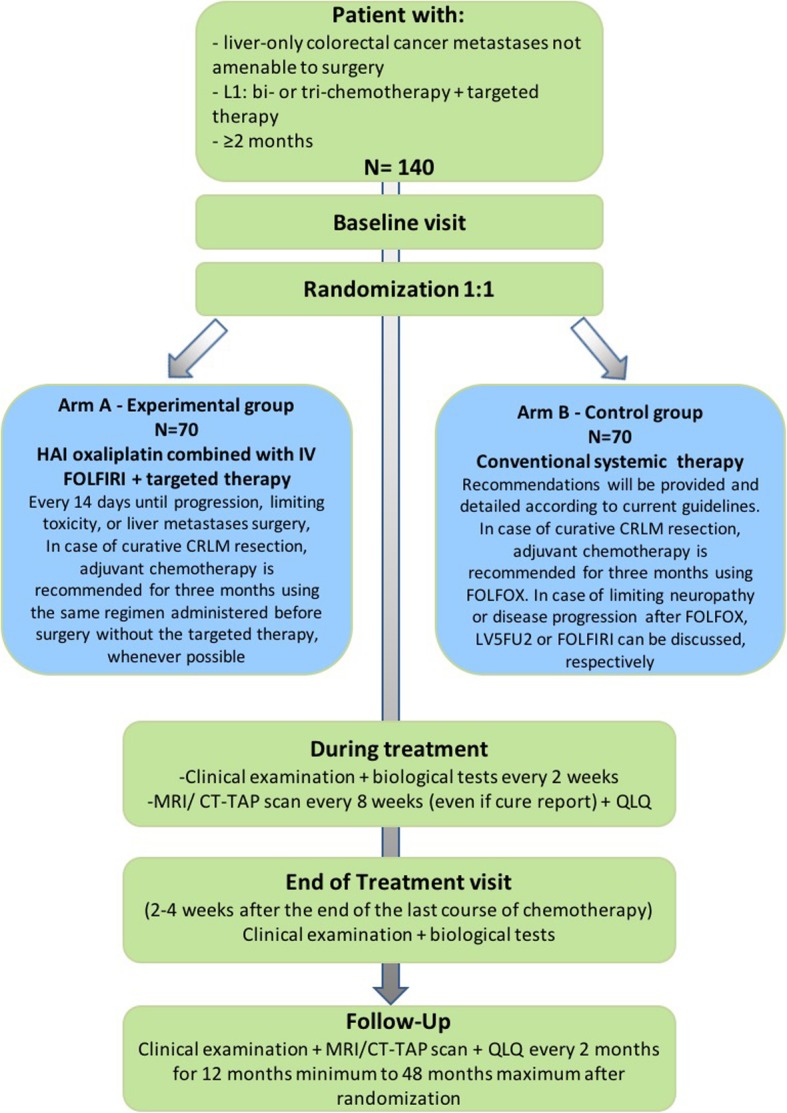
Flow diagram of the SULTAN study. L1: line 1; HAI: hepatic arterial infusion; CRLM: colorectal liver metastases; MRI: Magnetic resonance imaging; CT-TAP: computed tomography of thorax, abdomen and pelvis; QLQ: quality of life questionnaire

This study is designed as a multicenter, randomized, comparative phase II trial. The study flow-chart is detailed on Fig. 1.

### Study objectives and endpoints

#### Primary objective

The primary objective is to assess the efficacy of HAI in terms of complete (R0-R1) resection (or ablation) rate (CRR) in patients with liver-only CRLM not amenable to curative-intent resection (and/or ablation) after at least 2 months of induction sys-CT. Randomized patients will receive either chemotherapy intensification combining HAI oxaliplatin plus systemic FOLFIRI and targeted therapy (i.e. anti-EGFR antibody or bevacizumab) or conventional sys-CT and targeted therapy (i.e. anti-EGFR or antiangiogenic antibody).

#### Secondary objectives

The secondary objectives include the efficacy of combined HAI and sys-CT on OS, progression-free survival (PFS; overall, hepatic, and extrahepatic), ORR, and depth of response (DoR). For this study, OS is defined by the delay between randomization and the occurrence of death due to any cause, or the date of last follow-up in patients alive. PFS is defined by the delay between randomization and the occurrence of the first progression whatever its time of occurrence or death, or the date of last follow-up in patients alive without progression. Progression will be defined according to response evaluation criteria in solid tumors (RECIST) v1.1. For hepatic PFS, only hepatic progressions are taken into account, patients with extra-hepatic progression are censored at the time of this progression. Extra-hepatic PFS is the opposite, only extra-hepatic progressions are taken into account, patients with hepatic progression are censored at the time of this progression. ORR is defined as the best overall response according to RECIST v1.1 evaluated by computed tomography of thorax, abdomen and pelvis (CT-TAP) scan or magnetic resonance imaging (MRI) every 8 weeks. DoR is defined as the relative change in the sum of longest diameters of RECIST target lesions at the nadir, in the absence of new lesions or progression of non-target lesions, as compared to baseline.

The feasibility of treatments, with the proportion of patients receiving ≥4 cycles of HAI oxaliplatin (experimental arm only), the dose-intensity of oxaliplatin and other systemic cytotoxic/targeted agents received, and the proportion of patients receiving ≥4 cycles of sys-CT (both arms) will also be evaluated.

Finally, the tolerance will be carefully considered using the National Cancer Institute - Common Terminology Criteria for Adverse Events (NCI-CTCAE) v4.0 to evaluate the toxicity of HAI and sys-CT, including HAI catheter-related complications and treatment related mortality.

### Ancillary objectives

The SULTAN study also encompasses ancillary evaluation of the duration of objectives and stable responses, the relative change in the sum of longest diameters of RECIST target lesions at week 8 compared to baseline, and the complete pathological response in case of CRLM resection. For the patients with curative surgery, time without any chemotherapy in both arms in the period between R0/R1 surgery and new chemotherapy for recurrence or date of last follow-up in the absence of recurrence will be evaluated. The European Organization for Research and Treatment of Cancer (EORTC) core quality of life questionnaire (QLQ) EORTC QLQ-C30 associated with the Liver colorectal metastasis Module QLQ-LMC21 will be used to evaluate the quality of life of patients included in this study [18].

### Study population

#### Inclusion criteria

Eligible patients should have a histologically confirmed stage IV CRC with a radiologic or histologic proof of CRLM not amenable to a curative intent-treatment after at least 2 months and no more than 6 months of first-line induction sys-CT with oxaliplatin and/or irinotecan combined with a fluoropyrimidine and a targeted therapy. Unresectability of the CRLM will be confirmed by a centralized multidisciplinary expert panel according the following criteria: upfront R0/R1 resection of all CRLM (that leaves at least two adequately perfused and drained segments) not possible, and/or metastases in contact with major vessels of the remnant liver which would require resection of the vessel for an R0 resection (i.e., tumor involvement of main portal right and left portal veins, of the three main hepatic veins, or of the retrohepatic vena cava), and/or documented progressive disease on imaging (according to the RECIST v1.1 criteria) or doubling of serum levels of tumor markers following ≥2 months of induction chemotherapy. Patients must be 18 years or older, have a good general health status, normal liver, kidney, cardiac, hematologic, and coagulation functions. Patients should also agree to contraceptive methods, be affiliated to a social security regimen, and provide a signed informed consent form before study entry (Table 1).

**Table 1:** List of eligibility criteria of the SULTAN study

Inclusion criteria	Exclusion criteria
1. Histologically confirmed CRC, and radiologic or histologic proof of CRLM not amenable to a curative intent-treatment after 2 months to 6 months of first-line induction chemotherapy 2. First-line chemotherapy with oxaliplatin and/or irinotecan combined with a fluoropyrimidine and a targeted therapy (e.g., anti-EGFR or antiangiogenic antibody) for metastatic disease (patients ending their adjuvant chemotherapy after primary tumor resection since more than 6 months should also have received first-line chemotherapy for metastatic disease) 3. Unresectability of the CRLM will be confirmed by a centralized multidisciplinary expert panel (composed of surgeons, radiologists, interventional radiologists and medical oncologists). The panel will review the CT scan and MRI of the patients (weekly web conference). Non-resectability criteria (one of the following criteria): ✓ Upfront R0/R1 resection of all CRLM (that leaves at least two adequately perfused and drained segments) is not possible ✓ and/or metastases in contact with major vessels of the remnant liver which would require resection of the vessel for an R0 resection (i.e., tumor involvement of main portal right and left portal veins, of the three main hepatic veins, or of the retrohepatic vena cava) ✓ and/or documented progressive disease on imaging (according to the RECIST v1.1 criteria) or doubling of serum levels of carcinoembryonic antigen (CEA) or CA 19.9 following ≥2 months of induction chemotherapy 4. At least one measurable liver metastasis according to the RECIST v1.1 5. Age ≥ 18 years 6. ECOG performance status 0–1 7. Normal liver function: bilirubin < 1.5 x upper limit of normal values (ULN), aminotransferases < 5 ULN, alkaline phosphatase < 5 ULN 8. International normalized ratio (INR) < 1.5 ULN 9. Neutrophils > 1500/mm^3^, platelets > 100,000/mm^3^, hemoglobin > 9 g/dL (transfusion allowed) 10. Calculated creatinine clearance > 50 mL/min (Cockcroft and Gault formula) 11. Informed consent signed by the patient or his/her legal representative 12. Patient affiliated to a social security regimen 13. Potentially reproductive patients must agree to use an effective contraceptive method or practice adequate methods of birth control or practice complete abstinence while on treatment, and for at least 6 months after the last dose of study drug	1. Patient eligible for curative-intent treatment of CRLM (i.e. resection and/or thermoablation), according to local multidisciplinary team and/or central review 2. Definitive anatomical contraindication to complete surgical resection: a. More than two lesions in all liver segments b. Bilobar liver metastasis and more than three lesions > 3 cm in the hepatic lobe the least affected (i.e. the future remnant liver) c. Bilobar liver metastasis and disease liver extend > 50% 3. Extrahepatic metastases (except ≤3 lung nodules < 10 mm deemed amenable to curative-intent resection/thermoablation and non-resected primary tumor with no or mild symptoms) 4. Patient with contraindication for trial drugs; contraindication limited to targeted therapy (e.g., anti-EGFR or antiangiogenic antibody) are allowed 5. Disease progression after FOLFOXIRI/FOLFIRINOX 6. Sensory neuropathy ≥ grade 2 (NCI-CTAE v.4.0) 7. If patients received bevacizumab, following non-inclusion criteria must be respected: a. Proteinuria > 1 g b. Gastro-intestinal fistulae or perforation c. Hypersensitivity to Chinese hamster ovary cell products or other human recombinant antibody d. Major surgery in the last 28 days 8. If patients received panitumumab, following non-inclusion criteria must be respected: a. Interstitial lung disease b. Pulmonary fibrosis 9. Significant chronic liver disease (resulting in portal hypertension and/or liver failure) 10. Allergy to contrast media that cannot be managed with standard care 11. Previous organ transplantation, HIV or other immunodeficiency syndromes 12. Concomitant or past history of cancer within 5 years prior to entry into the trial (except treated basal-cell skin cancer or in situ carcinoma of the cervix) 13. Patients with clinically significant active heart disease or myocardial infarction in the last 6 months 14. Concomitant medications/comorbidities that may prevent the patient from receiving study treatments as uncontrolled intercurrent illness (for instance: active infection, active inflammatory disorders, inflammatory bowel disease, intestinal obstruction, uncontrolled hypertension systolic > 15 and diastolic > 9, symptomatic congestive heart failure…) 15. Ionic disorders as: a. Kalemia ≤1 x ULN b. Magnesemia <0.5 mmol/L c. Calcemia <2 mmol/L 16. Patient with a dihydropyrimidine dehydrogenase deficiency (DPD): the test should be done for all patients before first 5-FU administration, according recommendations about the high risk of no testing DPD in patient before 5-FU administration 17. QT/QTc > 450 msec (men) and > 470 msec (women) 18. Concomitant intake of St. John’s wort 19. Patient already included in another clinical trial with an experimental treatment 20. Pregnancy or lactation 21. Patients deprived of liberty or under guardianship 22. Patients unable to undergo medical monitoring for geographical, social or psychological reasons

#### Exclusion criteria

Patients eligible for curative-intent treatment of CRLM, with definitive anatomical contraindications to complete surgical resection, with extrahepatic mestastasis or with disease progression after FOLFOXIRI/FOLFIRINOX treatment are not eligible for the SULTAN study. Other criteria comprise patients with contraindications to study drugs (contraindication limited to targeted therapy are allowed), concomitant medications/comorbidities that may prevent the patient from receiving study treatments, concomitant intake of St. John’s wort, or allergy to contrast media that cannot be managed with standard care. Specific contraindications to the administration of bevacizumab or panitumumab are also considered exclusion criteria. Patients with sensory neuropathy ≥ grade 2 (NCI-CTAE v.4.0), significant chronic liver disease, history of cancer within 5 years prior to entry into the study (other than adequately treated basal-cell skin cancer or in situ carcinoma of the cervix), clinically significant active heart disease or myocardial infarction in the last 6 months, risk of developing ventricular arrhythmia, previous organ transplantation, HIV or other immunodeficiency syndromes, or dihydropyrimidine deshydrogenase deficiency cannot be included in the study. Patients should not be pregnant or breast-feeding, already included in another clinical study with an experimental molecule, deprived of liberty or under guardianship, or unable to undergo medical monitoring test for geographical, social or psychological reasons (Table 1).

### Randomization

Randomization will be performed using the module of the eCRF / Ennov Clinical® software and will be stratified using minimization method according to the following factors:

- Prior adjuvant or first-line induction oxaliplatin-based chemotherapy
- Tumor response to induction sys-CT at the time of patient inclusion (objective response versus stable disease versus progressive disease)
- Center

### Treatment schedule

Patients will be randomly assigned in a 1:1 ratio to the experimental arm (Arm A) or the control arm (Arm B).

#### Arm a (experimental arm)

HAI oxaliplatin combined with systemic FOLFIRI plus targeted therapy every 2 weeks. Patients will receive 2-h HAI oxaliplatin 100 mg/m^2^, combined with systemic modified FOLFIRI regimen without 5-FU bolus (1.5-h IV Irinotecan 180 mg/m^2^, no IV 5-FU bolus, 2-h IV leucovorin 400 mg/m^2^ [200 mg/m^2^ in 2 h for racemic mixture if L-folinic acid], followed by 46-h IV 5-FU 2400 mg/m^2^) plus 2-h IV cetuximab 500 mg/m^2^ or 1-h IV panitumumab 6 mg/kg or 30-min IV bevacizumab 5 mg/kg according to *RAS* status and prior response/tolerance to induction sys-CT.

#### Arm B (control arm)

Sys-CT, combined with a targeted therapy (i.e. anti-EGFR or antiangiogenic antibody), defined by the investigator before randomization according to response to prior induction chemotherapy, toxicity and duration of the induction chemotherapy, RAS status and current guidelines/standard of care [35, 36]. In order to minimize the heterogeneity between the two treatment arms, priority should be given to a biweekly regimens compatible with tumor response evaluation each 8 weeks, including FOLFIRI, FOLFOX, FOLFIRINOX, or LV5FU2 combined with a targeted therapy (i.e. 3 weekly schedules of XELOX or XELIRI are not authorized). The choice of treatment regimen in the control arm will be discussed with the expert panel before randomization for each patient.

In the control arm, the intensification of the induction sys-CT, if needed, will be done at the time of randomization and not as a second step following a less intensive treatment.

In both arms, treatment will be administrated until disease progression, limiting toxicity, or CRLM surgery. A 3-month adjuvant chemotherapy is recommended in case of curative-intent CRLM resection: the regimen administered before surgery, but without the targeted therapy, will be used whenever possible in the experimental arm, and FOLFOX will be used in the control arm. Three months of adjuvant chemotherapy with LV5FU2 or FOLFIRI (especially in case of objective tumor response under preoperative FOLFIRI regimen) can be discussed in case of limiting neuropathy or disease progression after FOLFOX, respectively.

The HAI catheter will be placed before initiating treatment, percutaneously by interventional radiologist under fluoroscopic monitoring in order to allow perfusion of the all liver volume through a single catheter linked to an implantable port [33], or surgically in case of planned laparotomy. A digital subtracted angiography during injection of contrast medium through the HAI catheter port will be systematically obtained before treatment initiation, and then every two courses of HAI. HAI will be delivered if the control angiogram confirms the patency of the catheter and perfusion of the entire liver without any extrahepatic perfusion or leak. Only physicians and nurses familiar with the HAI technique will perform the HAI chemotherapy.

### Assessments and follow-up

During treatment period, a physical, biological, and paraclinical examination will be performed every 2 weeks (Table 2 and Fig. 1). In the experimental arm, a verification of the port-catheter will be performed by angiogram (or angioscintigraphy) prior to treatment initiation with HAI oxaliplatin then every 28 days (more frequently upon request if port-catheter dysfunction is suspected) during the treatment phase.

**Table 2:** Trial flow chart of the SULTAN study

VISITS	Screening after 2 to 6 months of CT	Baseline Within 21 days before randomization	Treatment period			End of treatment 2 to 4 weeks after the last administration of the study treatment	Follow-up Every 2 monthsfor minimum 12 months to 48 months after randomization
			Every 2 weeks	Every 28 days	Every 8 weeks		
Inclusion / non-inclusion criteria		x					
Signed informed consent form	x						
Randomization (R)		x					
Medical history and prior treatment history		x					
Central review (verification on the unresecability of CRLM)		x					
PHYSICAL EXAMINATION^a^							
Complete clinical examination & vital signs		x	x	x	x	x	x
Performance status (ECOG)		x	x	x	x	x	x
Toxicities/adverse events/signs and symptoms		x	x	x	x	x	x
Concomitant treatments		x	x	x	x	x	
PARACLINICAL EXAMINATION							
Thoraco-abdomino and pelvic CT scan and/or liver MRI		x			x		x
Angiogram or scintigraphic hepatic infusion in the experimental arm		x^b^		x^c^			
ECG^a^		x	x	x	x		
BIOLOGICAL TESTS^a^							
Hematology (neutrophils, platelets, haemoglobin),		x	x	x	x	x	
Biochemistry (including kalemia, magnesemia calcemia, glycemia)		x	x	x	x	x	
Liver function (alkaline phosphatase, total and conjugated bilirubin, AST, ALT, LDH)		x	x	x	x	x	
Albuminemia, Protidemia		x	x	x	x	x	
INR		x					
Renal function (creatininemia, urea, calculated creatine clearance)		x	x	x	x	x	
Proteinuria		x	x^d^	x^d^	x^d^		
Pregnancy test		x		x	x		
Tumor marker: CEA + CA 19.9		x			x		
QUALITY of LIFE QUESTIONNARY							
QLQ-C30 + QLQ-LMC21		x			x		x^e^

^a^within 7 days of randomization for baseline assessment and to be realized before and after oxaliplatin intravenous or intrahepatic arterial infusion (HAI); after randomization; ^b^after randomization and before the start of intra-arterial oxaliplatin; ^c^at least every 28 days during the treatment phase, more often if needed, ^d^only for patients who received bevacizumab, ^e^Until progression for a maximum 2 years

Every 8 weeks, a CT-TAP scans (and liver MRI if needed) will be performed and patients will be asked to complete the quality of life questionnaires.

A final evaluation will be realized within the 2 to 4 weeks following the last administration of the study treatment with physical and biological examinations.

In absence of progression, follow-up assessment will be performed every 2 months during minimum 12 months to 48 months after the randomization. For each visit, the assessments described in Table 2 will be performed. After progression, follow-up will only be performed every 2 months for a maximum of 48 months after the randomization and will consist in getting data about overall survival.

### Statistical considerations

#### Required number of patients

Based on the CRR as the primary objective of this comparative randomized multicenter phase II study, the hypotheses are the following:

- P0: conversion to resectability rate (i.e. complete (R0-R1) resection rate = CRR) in the control arm = 10%
- P1: conversion to resectability rate in the experimental arm = 30%

With these hypothesis and to have a 80% power (beta risk = 20%) and an alpha risk of 5% (two-sided Chi^2^ test), a total of 124 evaluable patients will be required (62 in each arm). Taking into account non-evaluable patients (primary failure of the HAI procedure in the experimental arm, patients receiving less than 4 cycles of HAI oxaliplatin) (at least 10%), additional patients will have to be randomized to ensure a sufficient number of evaluable patients in the HAI arm for the per-protocol analysis. The rate of patients receiving less than 4 chemotherapy cycles (i.e. around 2 months of treatment) is expected to be unequal between both arms (around < 5% in the control arm and around 20% in the experimental arm). Overall, 140 patients will have to be included.

Most data from the literature are derived from retrospective or prospective non-randomized studies. Results from randomized trials are scarce, and suffer from not well defined/debatable unresectability criteria for CRLM. It is expected that most of the resection will occur within 6 months after randomization. The 30% R0-R1 resection rate in the HAI arm (experimental arm) is based on the results of three prospective single-arm HAI studies using three cytotoxic agents (fluoropyrimidine + oxaliplatin + irinotecan) and a targeted therapy (cetuximab, panitumumab, or bevacizumab in two of these studies) [27, 32, 37]. The 10% R0-R1 resection rate in the control arm is not well documented in the literature, particularly since this study will include patients with miscellaneous tumor response to prior induction sys-CT regimen(s). Conversion to complete resection after second-line sys-CT has not been prospectively assessed. Taking into account that responders to induction sys-CT will only be included if liver metastases are considered unresectable after at least 2 months of induction chemotherapy (using predefined rigorous unresectability criteria), and that response rates in the second- and later-line settings are usually less than 30%, the rate of conversion to R0-R1 resection is expected to be low (less than 10%).

Since the hypotheses are difficult to define and given the exploratory nature of this hypothesis in the control arm, we plan to monitor the CRR during the study in order to adjust the hypothesis and increase the sample size, if necessary.

#### Statistical analysis plan

The main analysis will be conducted in the “intention to treat” population defined as all randomized patients. A second per-protocol analysis in patients receiving at least 4 chemotherapy cycles in both arms following by a tumor response evaluation will be conducted. Analysis will be performed with a minimum follow-up of 1 year.

Results for the primary and secondary endpoints will be presented by arm with a confidence interval at 95% (Rothman for survival data). Safety data will be reported according to their frequency and by system organ class. The rate of patients with at least one severe toxicity (grade > 3) and at least one clinically relevant severe toxicity will be presented. For overall survival, survival rates at 12, 24, and 36 months and median will be calculated. For hepatic and overall progression-free survival, rates at 6, 12 and 24 months and median will be calculated. Competing risk approach will be used to study hepatic and extra-hepatic progressions. Main analyses will be stratified on tumor RAS/BRAF status (wild type versus RAS or BRAF mutation) using stratified logrank or Chi^2^ test. Such analysis may be completed by multivariate analyses using logistic (in particular for the main endpoint) or Cox model, as appropriate, stratified on tumor RAS/BRAF status and adjusted on stratification factor in case of imbalance between arms and/or other prognostic factors. A sensitivity analysis without the patients with BRAF mutations will be performed if some of these patients are included. For quality of life, either change from baseline using generalized mixed model for repeated assessments or time to a clinical relevant change will be used depending of the number and pattern of data available. Decision will be taken before any analysis.

### Toxicity monitoring

Intensity of events will be estimated according to the NCI-CTCAE classification, version 4.0 (toxicity score grade 1 to 5). Catheter-related complications will be specifically evaluated.

The decision to reduce the doses will be based on the maximum toxicity observed during the rest period. The dose adjustments should be based on the most significant toxicity grades. If a patient has several types of toxicity, the administered dose will be the one with the least risk for the patient. Besides the specificity of the HAI administration route managed as described above, dose adjustment criteria for usual drug-induced toxicity will be the same as those used for the systemic administration of the FOLFIRINOX regimen [38]. Provided that the abolition of the IV bolus 5-FU in the HAI arm will prevent hematologic toxicity, G (M)-CSF will not be recommended as primary prophylaxis, but will be considered for high-risk patients. If abdominal pain is observed during or after HAI oxaliplatin, verification of port-catheter by angiogram will be performed systematically before the next administration. In case of documented extrahepatic perfusion and persistent abdominal pain, upper gastrointestinal endoscopy will have to be performed looking for a gastroduodenal ulcer.

## Discussion

Our trial is the first randomized trial aiming to validate the use of HAI chemotherapy as a salvage therapy in patients with liver-only CRLM. Given the long-term survival of patients downstaged from an initially inoperable to a resectable state (~ 50% for downstaged patients versus < 15% in non-resected patients at 5 years) [9], increasing CRR is a reasonable objective in patients with liver-only CRLM.

Despite a reproducible significant increase in tumor response rate obtained with HAI chemotherapy, one of the reasons why most previous randomized trials using HAI FUDR or 5-FU failed to provide significant increase in OS [23] is the lack of sys-CT in the HAI arm favoring extra-hepatic progression. Since the availability of “modern” sys-CT, no randomized HAI study was further performed. The available data in the literature and the observed CRR in previous studies [31, 32, 39] in patients with unresectable CRLM who received prior sys-CT are very encouraging and suggest that it could be interesting to evaluate chemotherapy intensification with HAI oxaliplatin, in order to increase the CRR and eventually PFS and OS.

Given the high ORRs obtained with current first-line systemic regimens in patients with CRLM, the intensified therapeutic strategy combining HAI and sys-CT may not be systematically proposed in the frontline setting; nonetheless, it should be introduced relatively early in patients with insufficient tumor response after induction chemotherapy.

Catheter dysfunction and abdominal pain during infusion are the main described side-effects which can limit the feasibility of HAI oxaliplatin. Catheter dysfunction, include extra-hepatic diffusion of chemotherapy (which can often be managed by percutaneous embolization of hepatic collateral vessels) that may cause gastroduodenal ulcerations, or thrombosis of the hepatic artery/occlusion of the catheter which can be manage with careful thrombolysis. Abdominal pain during intra-arterial infusion, which seems to be a specific complication of HAI oxaliplatin can be managed with concomitant systemic analgesia.

Toxicity related to HAI chemotherapy may be prevented or managed by teams experienced in this route of chemotherapy. Our trial is conducted in carefully selected centers within the PRODIGE intergroup (UNICANCER GI, FFCD, and GERCOR). The coordinating center (Gustave Roussy) already trained many of these selected centers through dedicated workshops during the past 3 years. The workshops included HAI training courses (for oncologists, surgeons, radiologists, and nurses), live demonstrations in surgical and interventional radiology operative rooms, and interactive lectures/sessions.

Another aspect to insure the feasibility of the project is the centralized review to confirm the CRLM unresectability and other eligibility criteria before each patient inclusion.

Depending on the results obtained, this randomized Phase II study will be expanded into a Phase III study to demonstrate the superiority of a therapeutic intensified strategy based on HAI chemotherapy compared to sys-CT in patients with liver-only CRLM. If confirmed, this will have a clinically relevant impact on patient survival as well as on public health because i) CRC is one of the second most frequent cause of cancer-related death; ii) CRLM are the most frequent cause of death in patients with CRC; iii) resectability is the most important prognostic factor for overall survival in metastatic CRC.

The recruitment of patients in the SULTAN study is planned for 3 years. Currently, 11 patients have been screened and 7 of them have been randomized.

## Data Availability

Not applicable.

## Abbreviations

5-FU: 5-Fluorouracyl
CRC: Colorectal cancer
CRLM: Colorectal liver metastases
CRR: Complete resection rate
CT-TAP: Computed tomography of thorax, abdomen and pelvis
DoR: Depth of response
FUDR: Fluorodeoxyuridine
G (M)-CSF: Granulocyte-(macrophage) colony-stimulating factor
HAI: Hepatic arterial infusion
HIV: Human immunodeficiency virus
IV: Intravenous
MRI: Magnetic resonance imaging
NCI-CTCAE: National cancer institute - common terminology criteria for adverse events
ORR: Objective response rate
OS: Overall survival
PFS: Progression-free survival
RECIST: Response evaluation criteria in solid tumors
sys-CT: Systemic chemotherapy
ULN: Upper limit of normal
